# Biopolymer-Based Multilayer Capsules and Beads Made via Templating: Advantages, Hurdles and Perspectives

**DOI:** 10.3390/nano11102502

**Published:** 2021-09-26

**Authors:** Anna S. Vikulina, Jack Campbell

**Affiliations:** 1 Department of Theory and Bio-Systems, Max Planck Institute of Colloids and Interfaces, Am Mühlenberg, 1, 14476 Potsdam, Germany; 2 Bavarian Polymer Institute, Friedrich-Alexander University Erlangen-Nürnberg (FAU), Dr.-Mack-Straße, 77, 90762 Fürth, Germany; 3 School of Science and Technology, Nottingham Trent University, Clifton Lane, Nottingham NG11 8NS, UK; jack.campbell@ntu.ac.uk

**Keywords:** polyelectrolyte multilayers, encapsulation, calcium carbonate, drug delivery, shrinkage

## Abstract

One of the undeniable trends in modern bioengineering and nanotechnology is the use of various biomolecules, primarily of a polymeric nature, for the design and formulation of novel functional materials for controlled and targeted drug delivery, bioimaging and theranostics, tissue engineering, and other bioapplications. Biocompatibility, biodegradability, the possibility of replicating natural cellular microenvironments, and the minimal toxicity typical of biogenic polymers are features that have secured a growing interest in them as the building blocks for biomaterials of the fourth generation. Many recent studies showed the promise of the hard-templating approach for the fabrication of nano- and microparticles utilizing biopolymers. This review covers these studies, bringing together up-to-date knowledge on biopolymer-based multilayer capsules and beads, critically assessing the progress made in this field of research, and outlining the current challenges and perspectives of these architectures. According to the classification of the templates, the review sequentially considers biopolymer structures templated on non-porous particles, porous particles, and crystal drugs. Opportunities for the functionalization of biopolymer-based capsules to tailor them toward specific bioapplications is highlighted in a separate section.

## 1. Biopolymer-Based Multilayers

### 1.1. The LbL Technique

**Figure 1:**
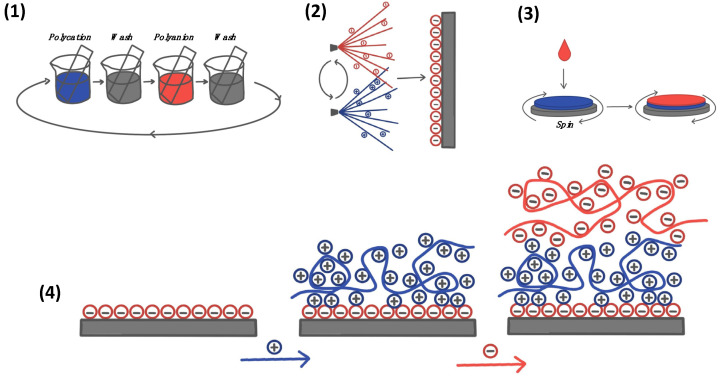
Schematic of classical approaches to form LbL films: (**1**) dip-coating, (**2**) spray-coating, and (**3**) spin-coating; (**4**) schematic of the build-up of LbL films upon a negatively charged substrate. Blue: polycation, red: polyanion. Adapted from reference [21].

With the introduction of the versatile approach of the layer-by-layer (LbL) assembly of oppositely charged polyelectrolytes some decades ago, the research field has grown exponentially, owing to the ease of control over the layered film properties (i.e., film thickness, homogeneity, stability, porosity, etc.) [1,2,3,4,5,6,7,8,9,10] and the ability to coat surfaces of varying geometries [11,12,13,14]. The LbL approach is based upon the build-up of oppositely charged polyelectrolytes, resulting in the build-up of polyelectrolyte multilayer (PEM) films, as illustrated in Figure 1(4) [15,16,17,18]. The capability to fine-tune their properties at both the macro- and nano-scale [19,20,21,22] has since spurred the rapid development of biocompatible materials for use in a plethora of bioapplications for both 2D (e.g., implant coatings [23,24], bio-scaffolds [25,26], biosensors/electronics [27], cell patterning [28] etc.), and 3D PEMs (i.e., polyelectrolyte multilayer capsules (PEMCs) for drug delivery [29,30,31] and tissue engineering [32] applications), including those used to mimic the cellular environment [33]. LbL assembly can be achieved by a variety of methods, including traditional dip-coating [34], spray-coating [35,36], and spin-coating [37] approaches (Figure 1(1)–(3)). More recent emerging methodologies include that of microfluidic assisted approaches via the 2D coating of channels and the packing of 3D capsules [32,38,39], as well as “brushing” approaches [19].

Interactions responsible for the build-up of these LbL films are numerous; the most common cause for the build-up are electrostatic interactions as, typically, oppositely charged polyelectrolytes are employed. Hydrogen bonding plays a significant role in multilayer formation, with some LbL films formed fully via hydrogen bonding interactions [40,41,42]. Hydrophobic interactions also play a role in the formation of films due to the hydrophobic nature of the hydrocarbon backbone of many of the polymers used [43], and they, therefore, cannot be neglected. Studies of the planar biopolymer-based LbL films demonstrated approaches to control the build-up of PEMs via temperature [44], ionic strength, and pH [45]. Covalent bond formation was manipulated to form LbL films via click-chemistry and they are much more robust in terms of withstanding drastic environmental change [46]. Free-standing 3D PEM structures were seen in recent work; biopolymer-based micro- and nano-capsules/gels are popular tools for the encapsulation and targeted delivery of fragile bioactives, including proteins [47], enzymes [48], and small molecules (such as ibuprofen [49], cisplatin [50], and polyphenol [51]). Polyelectrolyte multilayer capsules offer targeted delivery as well as a variety of release methods, including enzymatic degradation [52,53], magnetic field interactions [54,55], and ultrasound [56]. They are particularly attractive due to their tunable properties, whether via ionic strength, pH, or temperature, to control release dynamics and permeability [57,58,59,60,61].

### 1.2. The Classification of Sacraficial Templates and Issues of Biocompatability

A plethora of sacrificial templates are utilised to form such 3D structures and all host a variety of properties related to the final capsule structure, stability, and application. The variation in capsule internal structure is dependent on both the porosity of the template, as well as the size of the biopolyelectrolytes utilised for multilayer coating [62]. Templates may be categorized as porous, such as carbonates (i.e., calcium [63,64] and manganese [65,66] carbonates), mesoporous silica [67,68,69], and, potentially, calcium phosphate [70], or non-porous templates, such as polystyrene latex [71,72] and melamine formaldehyde [73]. Biological entities (e.g., erythrocytes [74,75] or bacteria (*Escherichia coli* (further *E. Coli*) for instance [76,77])) are also utilised as templates.

**Figure 2:**
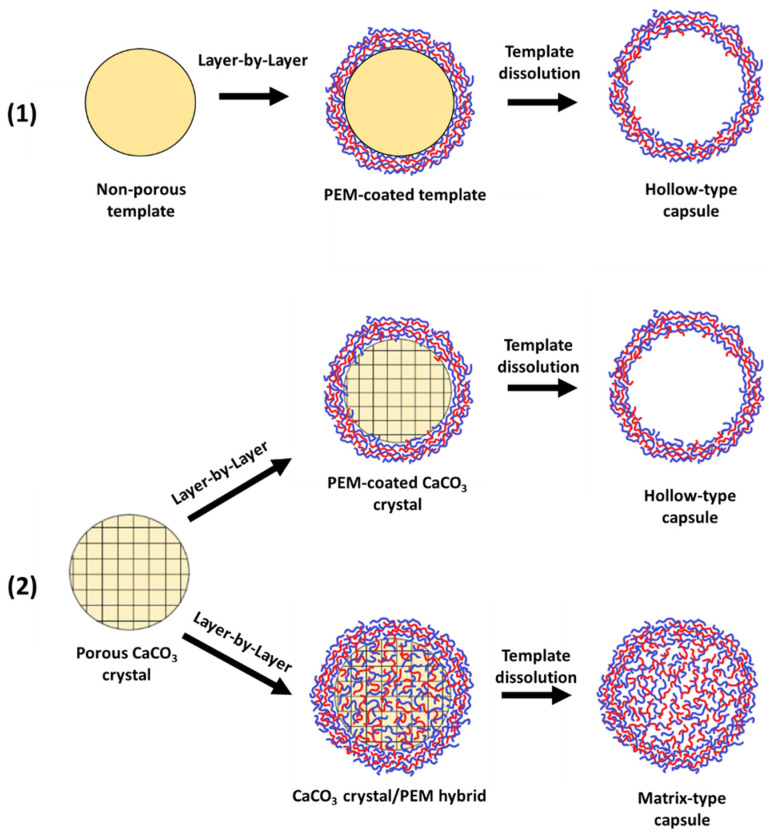
(**1**) The formation of hollow-type PEMCs upon a porous template. (**2**) The formation of hollow- and matrix-type PEMCs via either the formation of a polymer shell or CaCO_3_/PEM hybrid, respectively. (**2**) adapted with a permission from reference [79]. Copyright © 2021 American Chemical Society.

When utilizing a porous template, varying capsule structures are yielded. If the pore diameters are larger than that of the biopolymers used, during the initial deposition stages of LbL assembly the biopolymers adsorb to the template surface, however, in this case, the polymers are also able to permeate the template internal structure through surface pores and form an internal polymeric matrix [78]. Once the desired number of deposition stages is achieved, the template undergoes dissolution, leaving a polyelectrolyte multilayer capsule (PEMC) with an internal gel-like matrix; this capsule is coined as a matrix-type capsule, or a microgel [78,79,80] (Figure 2). During LbL deposition upon a non-porous template, the polyelectrolytes adsorb to the template surface and, following this, the template is eliminated, leaving a polymeric shell and a hollow lumen; these are known as hollow-type capsules. These shells may also form if no polymer is able to permeate the pores of a porous template, as illustrated in Figure 2, using vaterite CaCO_3_ as an example template. The terminology of such “matrix-type” capsules is largely in question; for instance, if less polymer is present in the capsule lumen compared to one completely filled, it is often overlooked due to its insignificance, and a suitable threshold between hollow and matrix should be established [79]. Moreover, the control over the capsule internal structure allows for control of the capsule release properties; a burst or sustained release can both be achieved with hollow- and matrix-type capsules, respectively [78].

For stable biopolymer-based capsules to form, a sacrificial core that undergoes dissolution at mild conditions, and gives rise to the minimal amount of osmotic pressure upon dissolution, is required to prevent the rupture of multilayers during dissolution. However, many sacrificial cores possess specific drawbacks typically related to dissolution conditions and their toxicity in vivo. For example, melamine formaldehyde (MF) cores undergo dissolution at low pH levels [81] and are, hence, not physiologically relevant, but upon dissolution there is an increase in osmotic pressure within the capsule, leading to high degrees of swelling and, sometimes, capsule rupture; if the capsule is able to form, residues of MF often remain within [82]. The carbonates MnCO_3_ and CaCO_3_ are particularly attractive; the vaterite polymorph of CaCO_3_ holds a well-developed porous structure [83], with pore sizes in the typical range of 5-35 nm, which can be further manipulated to control capsule internal structure [62]. It is biocompatible, low cost to produce [84], and undergoes dissolution at mild conditions [85], e.g., with ethylenediaminetetraacetic acid (EDTA) or citric acid, resulting in little osmotic pressure [83]. Importantly, biomacromolecules preserve their biological activities when encapsulated in the vaterite crystals [86,87,88].

A range of polymers were utilized to produce PEMCs and largely synthetic polymers [89,90]. Synthetic polyelectrolytes, despite their numerous advantages, including intrinsic robustness, large working windows of ionic strength and pH, as well as high levels of control over synthesis conditions (i.e., molecular weight), are typically toxic and have issues with biodegradability in the body [91,92]. As a result, there was a boom in the development of biopolymer-based materials for drug delivery and bioapplications in general. Biopolymers, however, are intrinsically liable and largely sensitive to their microenvironment, thereby making them onerous, but easily manipulated components to work with in the field of functional biomaterials [91,93]. Despite this slight drawback, they present numerous advantages due to their little-to-no toxicity, ease of biodegradability, and intrinsic biocompatibility [94]. Polysaccharides (typically glycosaminoglycans [95,96], functionalised chitosans [96,97], alginates [29,98], etc.) and polyamino acids (poly-L-glutamic acid (PGLU), poly-L-lysine (PLL), etc.) [30,99,100] are typically used for the formulation of 3D bio-based multilayer structures (i.e., PEMCs). Moreover, components of the extracellular matrix are also utilised, including, for instance, hyaluronic acid [101] and chondroitin sulphate [26,102]. With such biopolymers, we are able to exert control over the functionality of capsules, as well as achieve the mimicking of extracellular matrices and intracellular environment with ease in both 2D and 3D materials. This provides the opportunity to develop films and capsules to act as protein and bioactive reservoirs or anchors for encapsulation and cell adhesion/drug delivery [45,103,104,105,106]. Given the scope of biopolymer manipulation one can now achieve, the next sections will discuss the variety of sacrificial templates utilized for biopolymer-PEMC formulation and their specific drawbacks and advantages, along with the variety of PEMC functionalization performed within the literature.

## 2. Biopolymer-Based Capsules Templated on Non-Porous Templates

The advantages of the non-porous templates typically used to form PEMCs are the resultant properties following particle synthesis, including the range of controllable sizes available, starting from as small as tens of nanometres up to a few millimetres, and their typical monodispersity and stability as colloidal particles, which is ideal for PEMC formation and delivery carriers. Despite this, some of these carriers are hindered by their lack of biocompatibility both before and after template dissolution. For instance, non-porous silica templates require hydrofluoric acid to undergo dissolution, which is not ideal for biopolymeric materials.

DS/PR-based PEMCs were templated upon MF cores and, following treatment of MF with pH 1.7 HCl, MF residues were retained within the PEMC interior, as shown via Raman spectroscopy, likely due to DS-MF complex formation [82]. Although beneficial for the binding of encapsulated peroxidase, more biocompatible templates are preferred due to the harsh acidic removal conditions. The same was observed for ALG/CHI PEMs in the form of ALG-MF complexes and the binding of positively charged insulin at low pH levels [47], as well as within DNA/SP and ALG/PLL microcapsules [107]. The stable PEMC formation is dependent upon the osmotic pressure build-up during core dissolution and the ability of the MF oligomers to diffuse outwards [81], which may also be responsible for PEMC swelling during the core dissolution. Moreover, MF resin may become irreversibly adsorbed into the capsule shell and can contribute up to 20% of the capsule mass.

**Figure 3:**
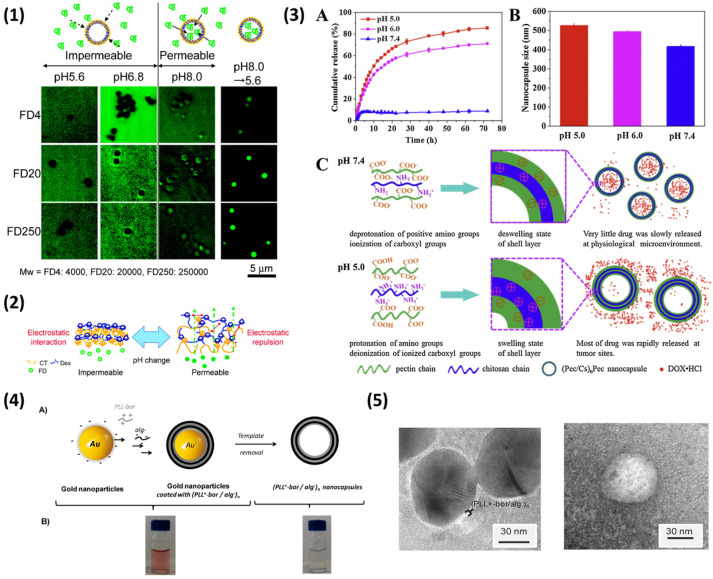
(**1**) Schematic and confocal microscopy images of the relative permeability of dextrans of varying molecular weight; FD4–4000, FD20–20,000, and FD250–250,000 in buffer solutions (pH 5.6, 0.05 M acetic acid buffer; and pH 6.8 and 8.0, 0.05 M TRIS buffer) containing dissolved fluorescein isothiocyanate-labelled dextran. A (**2**) schematic illustrating the change in permeability within the PEM when changing pH. Reprinted with permission from reference [110] copyright © 2008 American Chemical Society. The (**3**) cumulative release of DOX from (pectin/CHI)_3_/pectin nanocapsules (**A**), the hydrodynamic size of nanocapsules at varying pH (**B**), and the schematic illustration of the pH responsive nanocapsules (**C**). Reprinted with permission from reference [113] copyright © 2017 Elsevier. The (**4**) schematic illustration of the formation of glucose-responsive nanocapsules (**A**), and the suspensions of coated-gold nanoparticles (**left**) and nanocapsules following removal of the core (**right**). (**5**) TEM micrographs of (**left**) (PLL-bor)/alg−)4-coated gold nanoparticles and (**right**) nanocapsules. Reprinted with permission from reference [114] copyright © 2019 Elsevier.

Further to this, polystyrene latex (PS) templates were also employed for the formation of biopolymer PEMCs (fucoidan (FC)/CHI, for instance); the templates were treated with tetrahydrofuran (THF) [108] for two hours for removal and the PEMCs shrank by almost 50% from their template size. Of note, calcination of the coated PS core is also used for template removal [71], which is not ideal for many sensitive biogenic capsules. As PEMs formed on smooth non-porous templates are typically thinner and well defined [109], the loading and release of bioactives post-template dissolution can be well controlled via alterations in pH, ionic strength, and cross-linking of the shell. For instance, silica-templated DS/CHI PEMCs demonstrate a reversible permeability phenomenon; PEMCs were impermeable to dextrans from 4000–250,000 Da at pH < 6.8, but were permeable above pH 8. These permeable capsules were then reduced to pH 5.6 and the dextran was entrapped within the capsule interior (Figure 3(1)) [110].

This is attributed to the electrostatics of the biopolymers within the PEMs; at higher pH levels, there is an increased repulsion between the sulfonate groups upon DS and a reduced cationic charge on CHI, thus causing the polymers to change their conformation and increasing PEM permeability [110], as illustrated in Figure 3(2). Similar behaviour is observed with HS/PR [111] and pGLU/CHI [112] PEMCs, as well as Pectin/CHI PEMCs with the release of DOX; DOX is not released at pH 7.4, but is rapidly released at pH levels 5 and 6. This was attributed to the swelling of the PEMCs upon decrease in pH due to the increased protonation state of CHI below pH 6.5 (pKa value), with a size increase of 418 to 527 nm from pH 7.4 to 5, respectively [113]. A schematic of this is shown in Figure 3(3).

This pH-responsive property is useful as a way to control the release of the bioactive encapsulated, especially in-terms of low intracellular or tumorous pHs in which the PEMCs may be uptaken. Further to this, metal nanoparticles may also be used as PEMC templates. Gold nanoparticles in particular were utilised as templates for glucose-sensitive ALG/phenylboronic-modified PLL PEMCs; the gold core was removed via the addition of potassium cyanide, followed by dialysis for the removal of the gold complex formed (the authors confirmed the lack of gold via inductively coupled plasma analysis) [114], as demonstrated in Figure 3(4),(5). The use of such well-established monodisperse metal nanoparticles as templates allows for the formulation of PEMCs on the nanoscale (~40 nm in this study). Further examples of non-porous templates utilised for the formation of biopolymer PEMCs can be found in Table 1.

However, a disadvantage of these non-porous templates is related to the bioactive loading capacity of the final PEMC formed. Only post-loading approaches may be applied to such PEMCs, which may hinder the relative amount of bioactive material encapsulated. Porous templates, for instance, can be pre-loaded prior to PEMC formation and their loading potential is greatly increased compared to non-porous templates. These templates will now be discussed.

## 3. Biopolymer-Based Capsules and Beads Templated on Porous Templates

### 3.1. Capsules Formed from Porous Templates

The use of porous templates (i.e., MnCO_3_, CaCO_3_, and MS) for the formation of PEMCs holds numerous advantages over that of non-porous templates. One of the most prevalent being the ability to load bioactive material into the template’s intrinsic porous structure. Porous vaterite CaCO_3_ has demonstrated enormous loading capacities [11,119,120,121,122]; lysozyme, for instance, can be loaded up to 500 ± 128 mg/g CaCO_3_ within sub-micron crystals [123], and superoxide dismutase can reach up to 240 ± 8 mg/g CaCO_3_ in crystals of ~4 μm [124]. Moreover, the loading of such bioactives may be enhanced via the pre-encapsulation of a polyelectrolyte of affinity to the material of interest. Shi et al. (2018) [125] enhanced the loading of lysozyme by the co-synthesis of heparin sulphate (HS) prior. Of great interest, this can also be applied to the encapsulation of low molecular weight bioactives, which are difficult to encapsulate due to their small size in comparison to the large pore sizes of such templates [126]. This was applied with lentinan [127] with DOX, and CMC [128] with daunorubicin. Balabushevich et al. (2019) [120] successfully encapsulated DOX via the electrostatic binding to a gel-like mucin matrix pre-encapsulated within the vaterite crystal, reaching doxorubicin content of up to 1.3 mg/g CaCO_3_. This provides scope for a variety of clinically-relevant drug delivery applications and the potential use of vaterite as vehicles with mucoadhesive properties [11,129]. Moreover, due to the variety of loading mechanisms, one is able to tailor the loading technique to the biomaterial of interest; for instance, certain macromolecules or nanoparticles may be sensitive to salt solutions forming the crystal template and, hence, adsorption may be preferred. In addition, freezing-induced loading was recently demonstrated with the loading of TiO_2_ nanoparticles [130].

**Figure 4:**
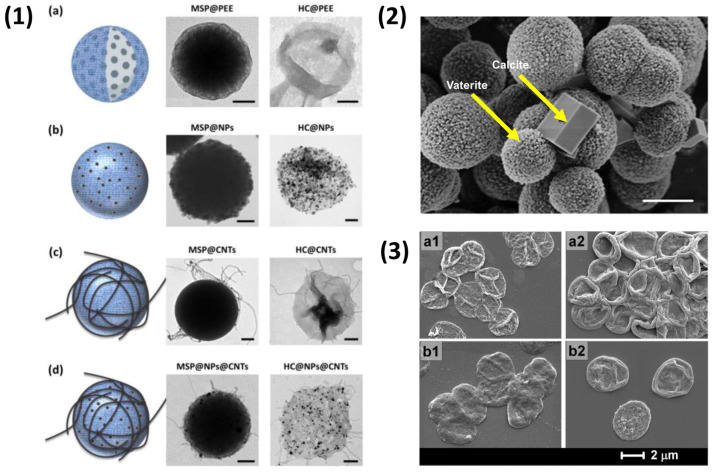
(**1**) Illustrations and transmission electron microscopy images of polyelectrolyte capsules before and after silica core dissolution. Hollow-capsules (**a**), CoFe_2_O_4_ NP-functionalised capsules (**b**), carbon nanotube (CNTs)-functionalised capsules (**c**), and capsules functionalised with both NPs and CNTs (**d**). Reprinted with permission from reference [67]. (**2**) Typical morphology of CaCO_3_ vaterite and calcite crystals via SEM, indicated by yellow arrow. Adapted with permission from reference [124] copyright © 2019 Elsevier. (**3**) SEM images of empty PEMCs, (DS/pARG)_3_–a1 and (ALG/pARG)_3_–b1, and post-loaded capsules with TRITC-BSA (DS/pARG)_3_–a2 and (ALG/pARG)_3_–b2. Reprinted with permission from reference [141] copyright © 2010 American Chemical Society.

The encapsulation of large sensitive macromolecules is possible due to the ability to form and load vaterite at close-to-physiological conditions [124], thereby maintaining the bioactivity of such materials [88]. Vaterite CaCO_3_ also proved itself a diverse material as both a stand-alone drug delivery vehicle [131,132,133,134] and as a material for surface coatings [123] for functional use as an antimicrobial carrier, for instance [135,136]. However, there can be an issue with the aggregation of vaterite CaCO_3_ crystals, making it difficult to form monodisperse templates without the need of additives, such as polypeptides [137]. Moreover, upon introduction of vaterite CaCO_3_ to aqueous solutions, the transformation from the porous vaterite polymorph to the non-porous calcite polymorph can take place over the course of hours or days. This means that bare-vaterite should be handled quickly when in the presence of water [138], however, polyelectrolyte coatings and stabilizers can prevent this [139,140]. This, however, can be beneficial for the release of encapsulated cargo via a re-crystallisation mechanism [120,124]. Despite this, due to its low cost production ($0.2–0.4/g dried weight), potential ease of scalability [85], and soft dissolution conditions, vaterite CaCO_3_ presents itself as an attractive template for PEMCs, the morphology of which is presented in Figure 4(2) with example biopolymer PEMCs below. Alternate templates, such as MS, for example, although advantageous with regard to pre-loading capability, are typically removed via hydrofluoric acid, much like non-porous silica, which means they are not suitable for bioapplications nor ideal for pre-encapsulated bioactives. Recently, however, PEMCs were formed via the removal of MS at physiological conditions via dissolution in a buffered salt solution [67], as demonstrated in Figure 4(1,3). Due to these advantageous properties, many more examples of biopolymer-based PEMCs are emerging, examples of such can be found in Table 2.

**Figure 5:**
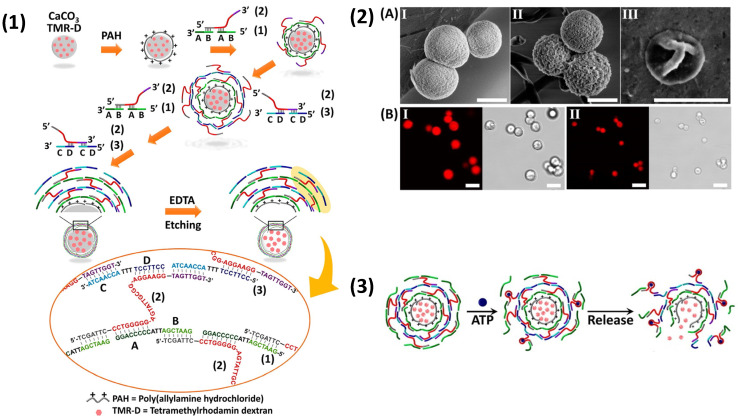
(**1**) Schematic illustration of the preparation of DNA microcapsules via CaCO_3_ templating. ATP-binding aptamer sequences, labelled in red colour, are embedded into DNA films as stimuli-sensitive switches. (**2**) SEM images of uncoated (**A**, I), DNA-coated (**A**, II) CaCO_3_ vaterite crystals and DNA PEMCs following EDTA addition (**A**, III). Below are corresponding confocal and brightfield confocal images of DNA-coated crystals (**B**, I) and DNA PEMCs (**B**, II). (**3**) Representation of ATP-induced PEMC rupture and release of TMR-D. Reprinted with permission from reference [164] copyright © 2015 American Chemical Society.

Apart from typical polysaccharide/amino acid PEMCs, vaterite CaCO_3_ and MnCO_3_ were also used as a template for DNA-based capsules. Tetramethylrhodamine-modified dextran (TMR-D)-co-synthesised vaterite crystals were coated with a primary layer of positively charged poly(allylamine hydrochloride) (PAH), followed by the LbL-build-up of two hybrid nucleic acids (for full sequences see [164]), of which one sequence contained the anti-adenosine triphosphate (ATP) aptamer. Following addition of EDTA and subsequent dissolution of CaCO_3_, hollow aptamer-cross-linked capsules were formed (Figure 5(1,2)) and underwent a shrinkage phenomenon (~3.2 um to ~2.5 um) [164], which was noted in other bio-capsule systems [79,150,162]. TMR-D was then subsequently released via the exposure of the capsules to ATP, which complexes with its aptamer, disrupting the bridging of DNA within the layers (Figure 5(3)). CaCO_3_-templated DNA-based capsules with size-selective macromolecule permeation were also produced using the LbL approach; 56 kDa dextran permeated the capsules, while 155 kDa dextran was inaccessible to the capsule [165,166]. Such DNA-based systems may prove useful for the size-selective encapsulation of macromolecules, as well as potentially low molecular weight drugs and genetic material [167,168].

**Figure 6:**
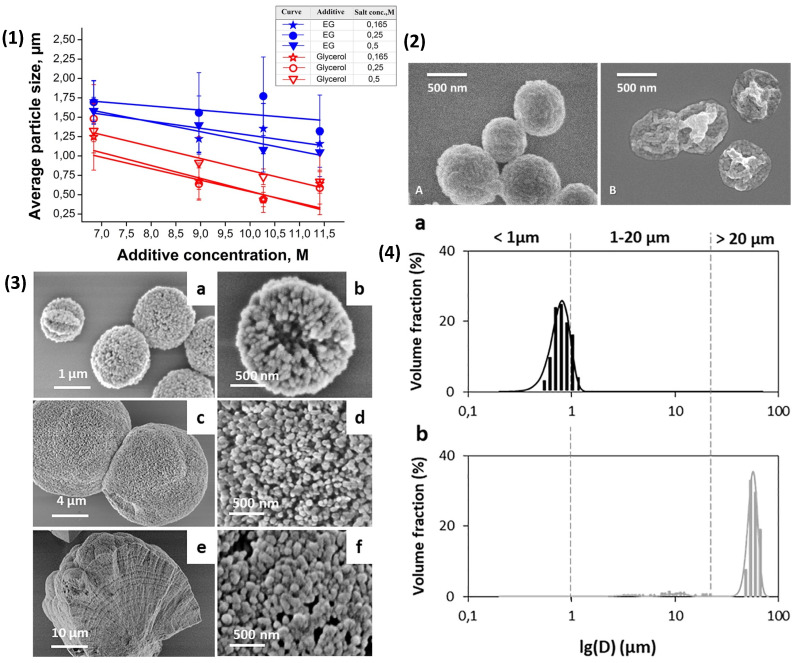
(**1**) Graphical representation of the effect of additive concentration on the size of vaterite CaCO_3_ crystals at different salt concentrations. (**2**) SEM images of sub-micron CaCO_3_ coated with (pARG/DS) 4.5 layers (**A**) and hollow PEMCs (**B**). Reprinted with permission from reference [84] copyright © 2016 American Chemical Society. (**3**) SEM images of vaterite CaCO_3_ sub-micron crystals (**a**,**b**), middle-sized CaCO_3_ crystals (**c**), and their typical surface (**d**). The spall of sub-millimetre vaterite CaCO_3_ crystal (**e**), and typical surface (**f**). (**4**) Typical size distribution of sub-micron-CaCO_3_ (**a**) and giant CaCO_3_ (**b**) crystals grown by the mixing of CaCl_2_ and Na_2_CO_3_ salts in water. Bars represent experimental data; lines show the fitting with Gaussian function. Reprinted with permission from reference [123] copyright © 2021 Elsevier.

The formation of nano-sized PEMCs, formed upon porous templates with varying biopolymers, can still be challenging as the typical size of CaCO_3_ PEMC templates fall within the range of 3–10 μm (Table 2). However, recent progress was made in the formulation of vaterite CaCO_3_ templates in the sub-micron and nano-regions [84,123,169,170]. Both additive [170] and additive-free [123] approaches emerged as facile methods to synthesise sub-micron vectors. For instance, when using a glycerol/gelatin formulation with ultrasonic treatment of the pre-cursor salt solutions, sizes of 54 ± 9 nm were achieved, along with a range of other sizes up to ~800 nm. Using additive-free methods, it is possible to reach sizes of close to 720 nm. Of note, we are able to reach sizes of up to 55 um CaCO_3_ crystals [123] for potential use as porogens [171] for tissue engineering scaffolds (Figure 6(3),(4)). Smaller sub-micron and nano-sized functional delivery vehicles are necessary for the effective treatment of ailments, including cancer, in which the enhanced permeation and retention effect is prevalent. Approaches concerning the shrinkage of PEMCs, with regard to reaching the necessary sizes for advanced drug delivery, are now emerging. Recent studies and their applications will be discussed in the next section.

#### Shrunken Biopolymer-Based Capsules

**Figure 7:**
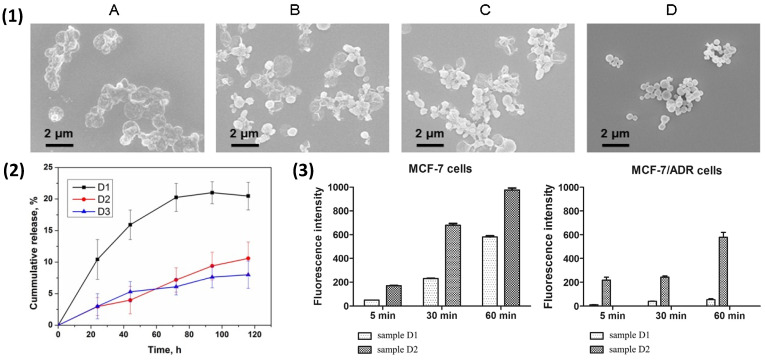
(**1**) SEM images of (Parg/DS)_4.5_ PEMCs: freshly prepared (**A**) and after the heat treatment at 50 °C for 15 min (**B**), at 50 °C for 120 min (**C**), and at 90 °C for 60 min (**D**). Reprinted with permission from reference [172] copyright © 2018 Elsevier. (**2**) DOX release from intact (initial size: 550 nm, final size: 550 nm, D1) and shrunken (initial size: 550 nm, final size: 290 nm, D2; and initial size: 290 nm, final size: 290 nm, D3) PEMCs. (**3**) Uptake of DOX-loaded PEMCs by human breast adenocarcinoma MCF-7 cells (left) and DOX-resistant MCF-7/ADR cells (right) after 5, 30, and 60 min incubation. Reprinted taken with permission from reference [146] copyright © 2019 Elsevier.

The shrinkage of PEMCs formed upon vaterite CaCO_3_ templates was reported numerous times [58,79,146,149], typically via the thermal treatment of capsules [61,172,173]; The observed shrinkage of PEM films, especially within capsule systems, holds important applications in drug delivery. Controlled shrinkage allows us to readily tune the size of delivery vehicles depending upon the targeted area; for example, the delivery of nano-capsules to tumorous cells saw an increase in research as of late due to their biodegradability and their ability to host large amounts of biological cargo. For instance, DS/pARG capsules were seen in recent work regarding the shrinkage of PEM capsules. Trushina et al. [172,174] subjected DS/PARG capsules to heat treatment at different temperatures (up to 90^o^ C); the heightened temperature results in the shrinkage of nano-capsules by factors of up to 42%. This was due to the temperature-induced annealing of biopolymers forming a more compact capsule shell upon shrinkage. This compaction is presented in Figure 7(1), wherein the shell clearly becomes denser upon compaction (shown via TEM imaging). Furthermore, it was demonstrated that increasing the ionic strength to that of physiological systems (0.15 M NaCl) and subjecting the capsules to heat treatment causes the capsules to collapse after 30 min of incubation. The authors attributed this to the drastic effect of ionic cross-linking upon the increase in ionic strength, thereby inhibiting the shrinking capability of DS/PARG capsules.

These capsules were also utilised in the encapsulation of chemotherapeutic drugs, including gemcitabine, clodronate [175], and doxorubicin [146].The shrunken capsules held a significantly higher rate of cellular uptake in vitro, and both gemcitabine and clodronate reduced the viability of lung cancer cells and the tumour-promoting function of bone marrow-derived macrophages, respectively. Doxorubicin-loaded capsules showed a sustained release profile, as opposed to a burst-release system (Figure 7(2)), which is typically observed. This was attributed to the thicker capsule shell, slowing the diffusion of the drug through the polymeric network. Indeed, a thicker capsule shell may be compared to the internal structure of that of matrix-type capsules, which are known to alter the release profiles due to the dense polyelectrolyte network within the capsule lumen [78]. Moreover, shrunken doxorubicin-loaded (DS/PARG)_3_ capsules were shown to accumulate within human breast adenocarcinoma MCF-7 and MCF-7/ADR (drug resistant) cells and managed to overcome the drug resistance of MCF-7/ADR cells (Figure 7(3)). Such sub-micron capsules may prove useful for future chemotherapeutic applications by taking advantage of advanced PEM shrinkage via highly dynamic biopolymers [21].

**Figure 8:**
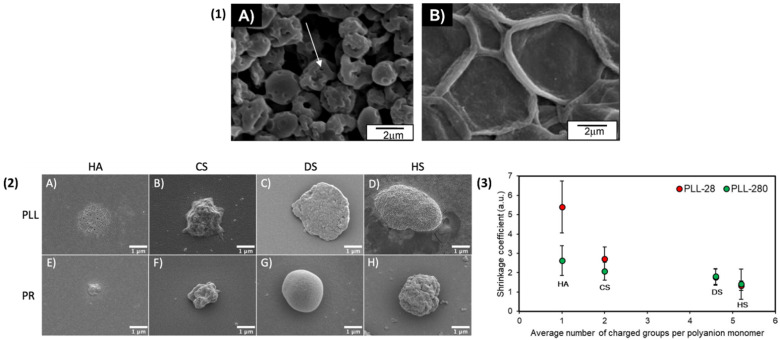
(**1**) SEM images of vaterite CaCO_3_-templated dried (HA/PLL) 4.5 PEMCs, the arrow indicates holes in the PEMC shell (**A**), and cross-linked via means of 200 mM EDC (HA/PLL) 4.5 PEMCs (**B**). Reprinted with permission from reference [149] copyright © 2010 American Chemical Society. (**2**) Typical SEM images of vaterite CaCO_3_-templated PEMCs consisting of 2.5 bilayers of PLL-based (top row) capsules consisting of HA, CS, DS, and HS are shown in images (**A**–**D**), respectively. PR-based (bottom row) capsules, consisting of HA, CS, DS, and HS, are shown in images (**E**–**H**), respectively. (**3**) Effect of number of charged groups upon the polyanion monomer unit on the shrinkage coefficient of PLL-28 (average molecular weight ~28 kDa) and PLL-280 (average molecular weight 280 kDa) PEMCs. Reprinted with permission from reference [79] copyright © 2021 American Chemical Society.

Biopolymer PEMCs with the ability to shrink at room temperature were also reported. Szarpak et al. (2010) [149] demonstrated the room temperature shrinkage of HA/PLL capsules upon dissolution of the CaCO_3_ core by ~50%, and demonstrated the inhibition of shrinkage upon cross-linking with EDC/NHS, as is demonstrated in Figure 8(1). This ambient shrinkage may potentially be used to entrap molecules of interest within the PEMC interior. Campbell et al. (2021) [79] reported the room temperature shrinkage (up to a factors of ~7) of a variety of PEMCs, as shown in Figure 8(2) (PLL and PR paired with HA/CS/DS/HS). The shrinkage was explained via the rearrangement of the biopolymers within the multilayers upon CaCO_3_ dissolution. Increasing the molecular weight of PLL resulted in a reduction in the shrinkage of PEMCs (Figure 8(3)), and a similar trend was observed upon the increase in polyanion charge density, giving scope for the facile control of final PEMC size depending upon the properties of the polymers used. Of interest, the majority of these PEMCs also adhere to the surface on which they are formed, allowing for the potential patterning of surfaces such as implants.

### 3.2. Beads Formed from Porous Templates

**Figure 9:**
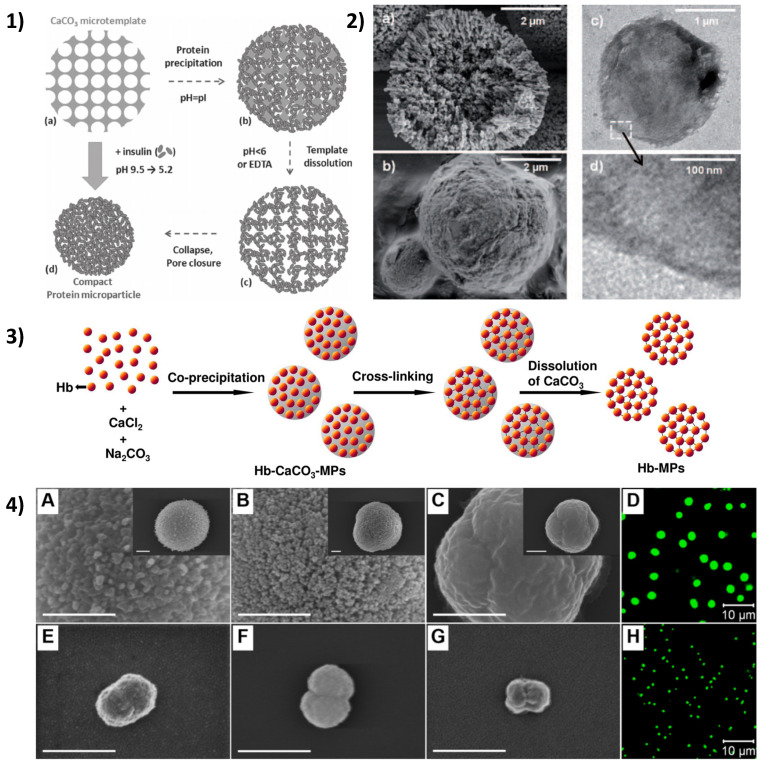
(**1**) Schematic of the formation of protein microparticles; a–b: the loading of porous CaCO_3_ templates with protein by isoelectric precipitation; b–c: dissolution of the CaCO_3_ template; and c-d: the shrinkage of protein matrix to a compact bead. Reprinted with permission from reference [122] copyright © 2012 John Wiley & Sons. (**2**) SEM images of vaterite CaCO_3_ crystals (**a**), and insulin microparticles (**b**). TEM images of insulin microparticle (**c**), and a magnified section in (**d**). Reprinted with permission from reference [185] copyright © 2010 John Wiley & Sons. (**3**) Schematic illustration of the production of haemoglobin microparticles. Reprinted with permission from reference [180] copyright © 2012 American Chemical Society. (**4**) SEM images of bare-CaCO_3_ crystal (**A**), haemoglobin-encapsulated CaCO_3_ crystal (**B**), and haemoglobin particle following CaCO_3_ dissolution (**C**). Corresponding confocal image of protein particles (**D**). SEM image of bare-MnCO_3_ particle (**E**), haemoglobin-encapsulated MnCO_3_ particle (**F**), and haemoglobin particle following MnCO_3_ dissolution (**G**). Corresponding confocal image of protein particles (**H**). Reprinted with permission from reference [181] copyright © 2018 Elsevier.

Simultaneously, the porous sacrificial templates were also utilized for the fabrication of polymer-based beads. Methodologically simplistic and robust, these particulate structures are formed via the loading of the respective molecule into a porous template via adsorption, followed the by cross-linking and elimination of this template, resulting in the formation of a pure polymeric particle within which is an inverted replica of the template utilized. Moreover, dependent upon the physiochemical properties of the biopolymer used and the extent of cross-linking, varying resultant properties in the final particles are seen, for instance, if strong inter-polymer interactions or a high degree of cross-linking occurs and free-standing porous particles are produced. Where there are weaker interactions, the particles may collapse to non-porous beads to minimize their contact with surrounding water [176]. Multicompartment heterogenous particles may also be produced [64]. Free-standing protein particles may be formed if the protein is insoluble at conditions where the template is soluble [176]. Through manipulation of the large loading capacity of vaterite CaCO_3_ crystals, insulin particles were formed. Insulin was incubated with CaCO_3_ crystals at pH 9.5, where CaCO_3_ is insoluble and insulin is soluble, and the pH was then slowly reduced down to pH 5.2. During this decrease, insulin solubility is reduced and precipitates within the crystal pores. At these lower pHs, CaCO_3_ also undergoes dissolution and pure insulin particles are formed (Figure 9(1)) [122,177]. Polyelectrolytes may also be deposited upon protein particles via LbL to control release, as with α-chymotrypsin for instance [178,179]. Haemoglobin microparticles were also formed, but via co-synthesis into CaCO_3_ crystals. Loading of the haemoglobin, followed by cross-linking with glutaraldehyde and dissolution of the carbonate template with 0.2 M EDTA (Figure 9(2)), resulted in smooth, spherical particles with an average diameter of 3.2 µm. These particles were successfully used as oxygen carriers, with each particle’s haemoglobin content consisting of at least one third of that found in an erythrocyte cell [180]. Successful fabrication of submicron haemoglobin particles was also achieved, via the use of peanut-shaped MnCO_3_ particles (Figure 9(4)) with very high haemoglobin uptake efficiency, utilising the same approach [181]. Alternatively to glutaraldehyde, particles of pure soy protein were formed via adsorption into CaCO_3_, utilising cross-linking with transglutaminase and dissolution of the template [182]. Polyelectrolyte-bridged protein particles were also templated upon mesoporous silica [183]. Moreover, dithioretinol was used to promote the opening of disulfide bonds of BSA entrapped within CaCO_3_ by co-synthesis and the subsequent removal of dithioretinol allowed for the formation of new disulfide bonds, both inter- and intra-molecularly, contributing to stable BSA particle formation [184]. UV-induced release of macromolecules from MnCO_3_-templated.

BSA particles can be achieved. The cross-linking of BSA with *ortho*-nitrobenzyl derivative 4-bromomethyl-3-nitrobenzoic acid under activation with 4-(4,6-dimethoxy-1,3,5-triazin-2-yl)-4-methylmorpholinium chloride, followed by MnCO_3_ dissolution, results in particulate BSA, which contains photo-cleavable C-N bonds, allowing for subsequent UV-controlled release of the cargo [186].

Recently, antibody-containing protein microparticles for the ELISA-based detection of human immunoglobulin G were produced by Neumann and Volodkin (2020) [187]. BSA and goat anti-human immunoglobulin G were co-synthesised into CaCO_3_ crystals, reaching loading efficiencies of up to 70%, followed by cross-linking with glutaraldehyde and dissolution with EDTA. Due to hydrophobic effects, the final porous protein particles shrank by 31% of the original crystal size, similarly to that of other protein-based particles [122,182]. With increasing control over the original porous template size (e.g., CaCO_3_, both with [170] and without [123] additives, MnCO_3_ [188], and mesoporous silica [189]), one may exert fine control over the final polymer particle size. Furthermore, through the use of thermo-responsive polymers, temperature-controlled shrinkage/swelling of pure polymeric particles can be achieved [190]. Recently reported, through the use of vaterite-templated poly(N-isopropylacrylamide) microgels, the temperature-controlled release of macromolecular drugs was demonstrated [191]. Ionic strength- and pH-induced swelling/shrinkage was also reported in gelatin-based microgels templated upon CaCO_3_ crystals [192]. Furthermore, cross-linked enzyme aggregates (CLEAs) may also be fabricated from this hard-templating approach via the immobilisation of the enzyme [193] within porous particles. CLEAs are popular materials in the field of biocatalysis as reusable catalysts [194], and the control of final aggregate size and porosity are important for the relative catalytic activity and practical use in industrial catalysis. Hard-templating may provide an appropriate means of controlling these properties [195,196,197].

## 4. Soft-Templated Biopolymer-Based Capsules

**Figure 10:**
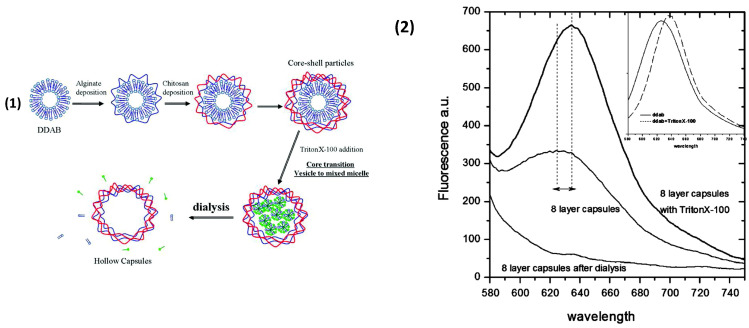
(**1**) Schematic illustration of the formation of liposome templated PEMCs. (**2**) Nile red fluorescence emission spectra (excitation at 530 nm) in 4 bilayer-coated liposomes (maximum at 625 nm) and following the addition of Triton X (maximum at 635 nm). Following dialysis, the fluorescence disappears. In the inset, a red shift for the bare DDAB vesicles is reported. The solid line refers to the fluorescence of Nile red in DDAB vesicles; the dashed line is the fluorescence spectra in mixed micelles. Reprinted with permission from reference [212] copyright © 2010 American Chemical Society.

Liposomes are spherical micro-/nano-structures that are formed from phospholipid bilayers with an aqueous compartment within. A plethora of lipids can be used to form liposomes; for instance, variations of phosphatidylglycerol (anionic at pH 7) and phosphatidylethanolamine (zwitterionic at pH 7), and phosphatidylcholine (zwitterionic at pH 7) (see review [198] and structures therein). Due to this, they possess amphiphilic character and can encapsulate hydrophilic molecules within their core and hydrophobic molecules within their lipid membranes. The lipid character of their structure gives them high biocompatibility and are, hence, very attractive as drug delivery vehicles due to their versatility. However, liposomes can possess poor stability unless in a buffered environment and can sometimes have poor drug-trapping potential, with some cargo elution [199,200]. This has led to extensive studies regarding the functionalisation of liposomes with different molecular species (i.e., antibodies, proteins, carbohydrates, and PEG) [201,202,203,204,205]. Moreover, multilayer coatings of biopolyelectrolytes to further protect the encapsulated cargo, during the delivery phase, and functionalise the outer-shell, have become attractive [206,207]; for instance, with coatings of PLL/pGLU [208], CHI with DS [209], and ALG [206], as well as protein-based BSA/lactoferrin [210]. A small number of studies report the soft-templating of biopolymer PEMCs upon liposomal structures. Cuomo et al. [211,212] demonstrated the formation of ALG/CHI PEMCs upon 300 nm phosphatidylcholine/ didodecyldimethylammonium bromide (DDAB) liposomes, a schematic of which is shown in Figure 10(1). The removal of the core was performed with a non-ionic surfactant (Triton X-100 in this study) via inducing a liposome-to-micelle transition, which was monitored with Nile red dye-sensitive to its microenvironment (whether a micelle or lipid bilayer; for example, fluorescence maxima in Figure 10(2)). Loaded liposomes may also be coated and immobilised onto functionalised surfaces (i.e., multilayer coated surfaces) to act as controlled delivery vectors [213,214].

**Figure 11:**
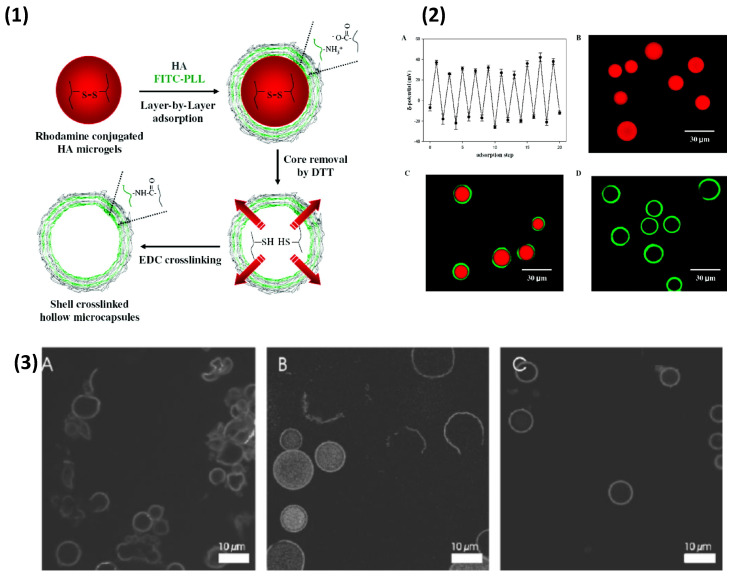
**(1)** Schematic illustration of the formation of cross-linked hollow HA/PLL PEMCs and (**2**) zeta potential as a function of layer number during LbL deposition upon HA microgels (**A**), confocal images of rhodamine-conjugated HA microgels (**B**), FITC-labelled HA/PLL shell containing rhodamine (**C**), and following core removal (**D**). Figures taken with permission from reference [217] copyright © 2007 American Chemical Society. (**3**) Confocal images of dex-HEMA microgels coated with C /pARG)_4_ (**A**), (pGLU(high molecular weight)/pARG)_4_ (**B**), and (DS/pARG)_4_ (**C**) after degradation of the microgel core. In (**A**), all microcapsules were broken and released their contents. In (**B**), both broken as well as intact (still filled with 150 kDa FITC-dextran) microcapsules were observed. The capsules in (**C**) remained intact, but had released their contents by diffusion through the bio-polyelectrolyte coating. Figure taken with permission from reference [216] copyright © 2007 John Wiley & Sons.

Further to liposomes, microgels may be used as soft templating materials, for instance dextran hydroxyethylmethacrylate (DEX-HEMA) microgels, which can be synthesised in a broad size range, with so-called giant microgels (150 μm) used for the formation of synthetic capsules [215]. Biopolymer-based PEMCs templated on DEX-HEMA were reported with pARG paired with CS, pASP, pGLU, and DS. The dissolution of the core microgel was performed with 0.1 M NaOH via hydrolysis of the cross-linking carbonate esters between dextran chains. Upon degradation, only pARG/DS produced stable hollow PEMCs, while the rest self-ruptured, which was attributed to the build-up of osmotic pressure within the polyelectrolyte shell; demonstrated in Figure 11(3). However, upon increasing the molecular weight of pGLU, a percentage of PEMCs remained intact (Figure 11), suggesting a larger polymer chain length may increase PEMC mechanical strength or permeability of the shell. Of note, HA, CHI, PLL, pONT, and ALG were tested as LbL components, but the authors reported instantaneous microgel aggregation upon dispersion into the biopolymer solution, whereas pARG did not cause this [216]. Disulfide-crosslinked HA gels (~16 μm) was also coated with HA/PLL multilayers, followed by the addition of dithiothretinol (at neutral pH), to cleave the sulfide linkages in order to remove the microgel core [217], as shown in Figure 11(1,2). Using such gels allows us to pre-encapsulate bioactive material pre-LbL deposition, much like that of porous inorganic templates (CaCO_3_/MnCO_3_/MS), and produce PEMCs under mild conditions suitable for biopolymers. Other potential soft materials may include that of PNIPAM and alginate gels [218,219].

Biological templates were also used as PEMC templates, with erythrocytes used in particular as templates for synthetic PEMCs [74,76]. It was reported that the removal of an erythrocyte core via NaOCl could lead to changes in the chemical nature of the polyelectrolytes used, with amino groups in PSS/PAH oxidised to nitro-, nitroso- and nitrile- groups, causing the subsequent cross-linking of PAH. Although advantageous in terms of mechanical stabilisation, this may not be suitable for such sensitive biopolyelectrolytes [220]. However, as of recently, live *E. coli* was used as PEMC template via the coating of ALG/CHI biopolymers, following dissolution via cell lysis (incubation in lysis buffer-(0.1% Triton X-100, 2 mM EDTA in 10 mM Tris-pH 8) with 100 μg/mL lysozyme overnight) [221]. Stable hollow PEMCs were formed with a slight increase in shell thickness, from 10–20 nm to 20–50 nm, which was attributed to alterations in polymer conformation on *E.coli* degradation. This demonstrated the novel formulation of biopolymer PEMCs on bacterial cells formed at soft conditions. This also leaves no harmful polymer residue in the shell, meaning it is suitable for release of cargo for biotherapeutic applications, perhaps opening up for a wider variety of biological PEMC templates.

## 5. Drug Crystal-Templated Biopolymer-Based Capsules

Key properties for the formulation of drug delivery vehicles include bioavailability, biodegradability, and high control over the drug content. A facile method to control drug loading is to use pure drug nano-crystals themselves. A number of approaches were successfully applied to reduce the size of such pure drug particles from the micro- to the nano-region, including both top-down [222,223] and bottom-up [224] approaches. The top-down approach involves the sonication or milling of coarse drug crystals to aid in the production of nano-sized crystals, whereas bottom-up involves the precipitation of nanocrystals from the dissolved drug via a solvent-induced supersaturation state, which is then followed by drug nucleation and subsequent growth. Precipitation may also be induced via pH change, if the drug of choice holds pH-dependent solubility, as well as via emulsification into organic solvent nanodroplets, in which nanocrystals may grow (for further details see chapter [225]). Many drugs, however, are typically poorly soluble in aqueous environments and, upon reduction of size, their solubility dramatically increases. This is due to the increase in the surface area to volume ratio of the crystal, giving increased solvent-crystal contact [226]. Despite this increasing drug bioavailability, such drug nanocrystals may solubilise and release drug molecules instantaneously at the target site or en route. Hence, LbL coatings present themselves as popular methods to control the drug release rate, as well as potentially the biodistribution if functionalised. Furthermore, one must be sure to deposit the polyelectrolytes at appropriate conditions, wherein the solubility of the drug is not increased nor decreased for the polyelectrolytes used, and retain their stability. Table 3 includes examples of biopolymer-coated drug nanocrystals.

**Figure 12:**
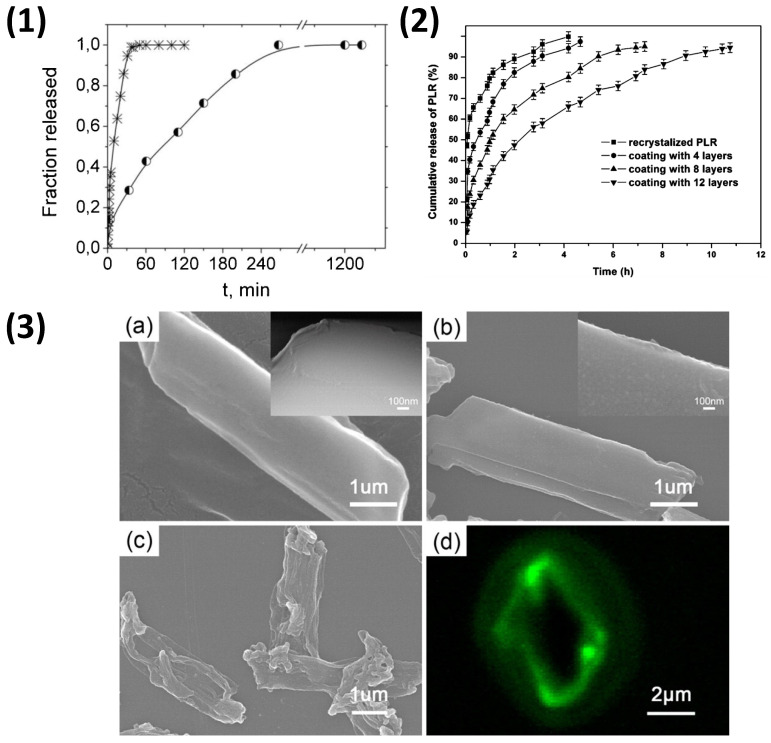
(**1**) Release profile of bare (*) and CHI/pectin coated (◐) indomethacin particles in phosphate buffer pH 7. Figure taken with permission from reference [230] copyright © 2017 Elsevier. (**2**) Release profiles of picloram from LS/CHI PEMCs with 0, 4, 8, and 12 polyelectrolyte layers, and (**3**) SEM images of recrystallised picloram (**a**), picloram coated with 5 bilayers (**b**), and hollow PEMCs after picloram release (**c**), with corresponding confocal image (**d**). Figures taken with permission from reference [235] copyright © 2013 American Chemical Society.

Nano-crystals of indomethacin, an anti-inflammatory drug, were prepared via the top-down approach using mortar and pestle grinding, followed by sonication. LbL coating of chitosan and pectin was performed at pH 4.5, where the drug is almost insoluble, and the CHI was fully dissociated, while the pectin was dissociated at 80%. Four layers were alternately deposited, and the subsequent release of indomethacin was studied at pH 7. The release was slowed for those drug crystals coated with CHI/pectin [230] (Figure 12(1)) to a saturation point within 5 h, compared to 1 h for uncoated crystals. This same effect was observed for picloram, a herbicide, coated with LS/CHI PEMs [235], (Figure 12(1)) as well as in SA/Gelitin, DS/Gelitin, and DS/CHI multilayers with naproxen crystals [229]. Furthermore, following the dissolution of the drug nano-crystal, a PEMC may remain if the shell has not ruptured. Following the dissolution of picloram, for instance, a hollow PEMC remains [235], as observed in Figure 12(3). Moreover, the coating of such nanocrystals with biopolymer PEMs is of particular interest due to their typically intrinsic biocompatibility and controllable permeability. Further, from drug crystals, those particles formed fully from bioactive material, such as the biopolymer beads, enzyme crystals [48,236], or protein aggregates discussed previously, are of interest to coat in order to control the release of protein or enzyme, as well as to protect such fragile cargo [178]. To ensure the safe travel of these bioactives to their target site, functionalisation of the PEMC shell can be of great importance and interest. This will be discussed in the next section.

## 6. Functionalisation of Biopolymer-Based Capsules

### 6.1. Functionalisation with Nanoparticles

The ability to functionalise the PEMC shell gives us the opportunity to form hybrid-PEMCs and tailor them toward specific bioapplications [237]. Functionalisation of PEM biocoatings with plasmonic nanoparticles was demonstrated as a powerful tool for controlled release of the payload [238] and for control over cell adhesion [239]. Functionalisation of PEMCs with nanoparticles serves similar purposes. Magnetic nanoparticles were embedded into the multilayer shell for increased accumulation at the target site, within DS/pARG capsules, for example [174,240]. For instance, Fe_3_O_4_ and doxorubicin-loaded PR-carboxymethylcellulose PEMCs showed greater efficacy against doxorubicin-resistant HeLa cells in mice, resulting in greater apoptotic effects in tumour cells. The authors attributed this to the controlled in vivo distribution of doxorubicin, which was controlled via an external magnetic field [241]. This gives scope for magnetic bio-capsules to be used in theranostics, both as drug delivery vehicles and contrast agents [240]. Superparamagnetic iron oxide nanoparticles were also adsorbed onto CaCO_3_-templated DS/pARG multilayer capsules through replacing a negatively charged layer. It was demonstrated the viability of HeLa and 293T cells was not altered after 24 h of exposure to the PEMCs. Following the application of a magnetic field, the phagocytosed capsules can be retained at the target site under physiologically relevant shear stress conditions, following HeLa-EGFP engulfment [242]. Nanoparticle functionalised PEMCs may also be used in photothermal therapy. Gold nanorods, encapsulated within GA-crosslinked CHI and ALG PEMCs, exhibit a photothermal effect, inducing a temperature increase and the collapse of the PEMC following near-infrared irradiation [243]. The authors attributed this to the thermal degradation of the aldehyde groups of glutaraldehyde, leading to multilayer instability. Of note, this system also acted as a dual-therapeutic agent with phototherapy combined with chemotherapy. The PEMC was also able to host doxorubicin (10.56% *w*/*w*), which was released upon the irradiation and subsequent collapse of the capsule.

X-ray radioprotective nanoceria was also previously incorporated into the polyelectrolyte shell of DS/pARG PEMCs. The PEMC acts to preserve the antioxidant effects of cerium and release it intracellularly in a controlled manner [244,245,246]. Cerium oxide acts as a reactive oxygen species scavenger and may also act as a “filter” within the PEMC shell, filtering out ROS and protecting its cargo, as shown through the protection of the encapsulated enzyme luciferase [247]. Furthermore, (haemoglobin/poly (ethylene glycol))_4_-coated CaCO_3_ crystals loaded with magnetic iron oxide nanoparticles showed an affinity for the adsorption of UO_2_^2+^, with the presence of the protein bilayers increasing percent uranyl sorption by ~90%. The authors proposed that this system may be used as an approach to separate damaging UO_2_^2+^ from contaminated bodies as a radioprotectant, due to the simple removal of the CaCO_3_ template and biocompatibility of the components used [248]).

SiO_2_-coated DS/pARG PEMCs were used as non-viral vectors for the delivery of CRISPR-Cas9 components, as the coated PEMCs act to protect the bioactive via greatly reduced shell permeability. Superior transfection was observed compared to commercial reagents and the authors demonstrated the ability of the CaCO_3_-templated PEMCs to overcome extra- and intracellular barriers to deliver genetic material [170,249]. Moreover, photoluminescent near-infrared emitting (750–1200 nm) MnCO_3_-templated PEMCs of ALG/PR with a capping layer DS and CHI were formed via the adsorption of Cd_x_Hg_1−x_Te nanocrystals into the polymer matrix. The authors proposed these PEMCs may be useful for the monitoring luminescence of the material in tissue as part of the drug delivery process [250]. Further to this, the incorporation of nanoparticles into such PEMs can result in the alterations of the mechanical properties. For instance, gold nanoparticles were shown to increase the Young’s modulus 16-fold in 2D HA/PLL PEMs [251], and increases in this value were also seen with silver nanoparticles and graphene flakes [252] as well as SiO_2_-coated PEMCs [249]. This holds positive implications in terms of tissue engineering applications with regard to the packing of biocompatible functionalised PEMCs together with varying surface properties and, hence, tunable cell adhesion and differentiation.

### 6.2. Functionalisation with Ligands and Antibodies

**Figure 13:**
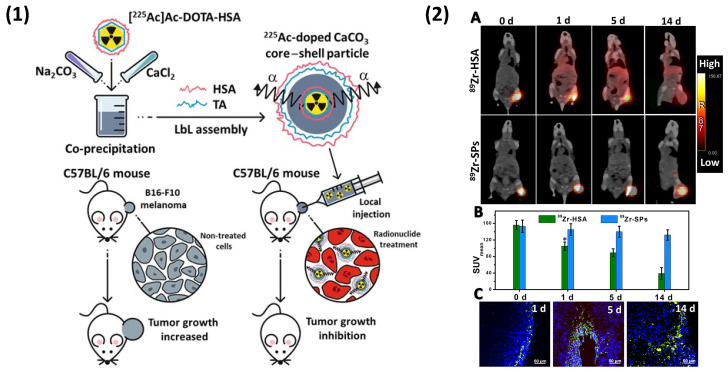
(**1**) Schematic illustration of the formation of sub-micron ^225^Ac-doped TA/HSA-coated CaCO_3_ particles and their application as local radionuclide therapy agents for melanoma treatment. (**2**) In vivo biodistribution studies after intratumoral injection of ^89^Zr-HSA and ^89^Zr-particles in B16-F10 melanoma-bearing mice: PET/CT images of mice treated with ^89^Zr-HSA (0.5 μCi, V = 50 μL) and ^89^Zr-particles (0.5 μCi, V = 50 μL, cSPs = 50 μg/mL, NSPs ~18 × 10^7^) at different time points (**A**). The biodistribution of radiolabelled test samples measured (standardized uptake value, SUV) (**B**). Confocal images of tumour tissue after intratumoral injection of FITC-labelled particles (blue colour corresponds to DAPI stained cell nuclei, and green colour to particles) (**C**). Reprinted with permission from reference [253] copyright © 2021 American Chemical Society.

**Figure 14:**
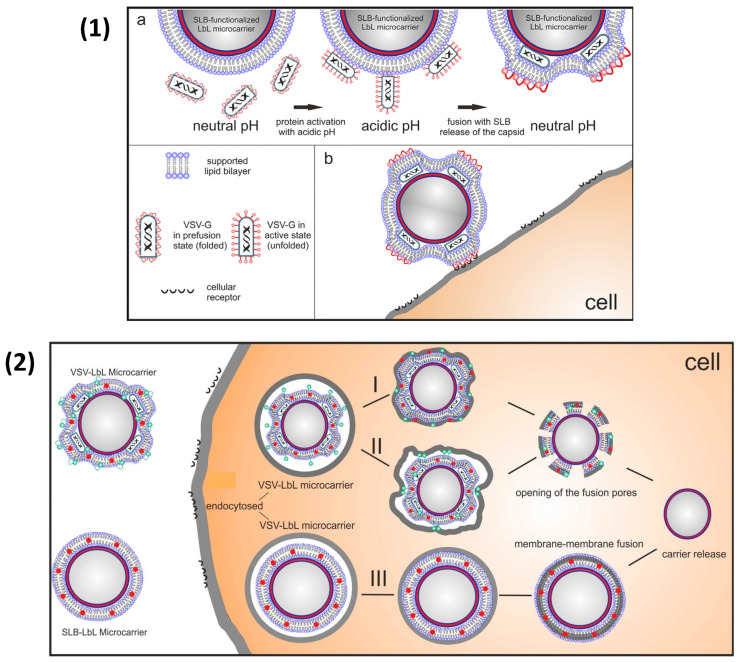
(**1**) Schematic illustration of the optimal virus assembly on lipid bilayer-coated -LbL microparticles using the unique conformational reversibility of the VSV-G protein. Assembly strategy is shown, making use of the unfolded state of the protein at pH 4, to initiate fusion with the lipid bilayer (**a**). After neutralization, VSV-G regains its prefusion state, allowing the protein to be available for specific cell interaction via receptor binding (**b**). Reprinted with permission from reference [256] copyright © 2018 American Chemical Society. (**2**) Schematic illustration of the possible fusion mechanisms between the lipid-bilayer-coated LbL coated particles and the endolysosomal membrane. In the upper panel, I and II, virus protein-mediated membrane fusion with endolysosome is shown, while the lower panel (III) illustrates the membrane-membrane fusion of nonfunctionalized particles and endolysosome. Red dots represent the fluorescent label of the lipid layer, and green dots demonstrate the formation of the fusion protein. Reprinted with permission from reference [255] copyright © 2020 American Chemical Society.

Alongside nanoparticles, LbL particles may be functionalised with certain molecules, or radiolabelled to either enhance the efficacy of the encapsulated agent, provide a means of in vivo imaging, and increase therapeutic effects, for example. Recently, biopolymer-coated CaCO_3_ particles were applied as local radionuclide therapy agents for melanoma treatment (Figure 13). Human serum albumin/TA-coated sub-micron CaCO_3_ particles were radiolabelled with actinium-225 to increase the efficacy of α-radionuclide therapy in mice bearing B16-F10 tumours [253], as well as zirconium-89 within the shell of PEMCs and coated-CaCO_3_ templates for the positron emission tomographic imaging of vehicles in vivo [253,254]. Figure 13(1) demonstrates a schematic for the radiotherapy treatment and subsequent tumour growth inhibition, while Figure 13(2) gives representative PET images of mice treated with ^89^Zr-labelled LbL particles, demonstrating the retained high resolution after 14 days [253]. The incorporation of biorelevant ligands to promote the targeting or cellular uptake of the PEMC is also of great interest. Scheffler et al. (2020) [255] demonstrated the assembly of the fusion protein (glycoprotein G) of the vesicular stomatitis virus (VSV-G) onto lipid bilayer coate SiO_2_ particles, pre-coated with DS/PR multilayers. The virus particles were isolated from the supernatant of infected BHK cells and, following UV inactivation, were incorporated onto the particle via the conformational reversibility and activity of the fusion protein in varying pH (Figure 14(1)) [256]. Fusion proteins mediate the entry of viruses into cells and were shown to enhance the uptake of these LbL particles in Vero cells compared to the PEM and lipid bilayer alone. Its potential pathing can be observed in Figure 14(2). This may also be applied to PEMCs with encapsulated cargo.

**Figure 15:**
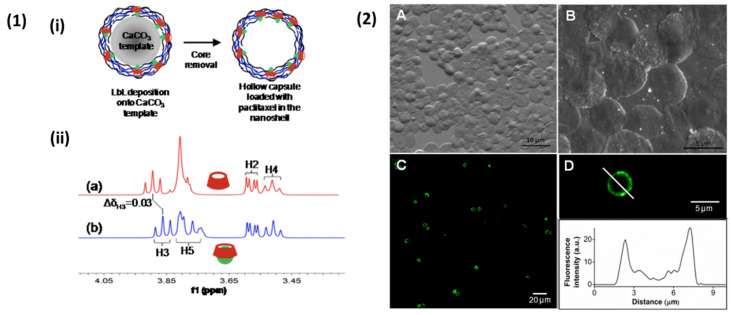
(**1**) Schematic illustration of the formation of cyclodextrin-modified HA/PLL PEMCs via dissolution of the CaCO_3_ template (**i**), the partial H NMR spectra of cyclodextrin (**a**), and mixture of cyclodextrin with paclitaxel (**b**) (**ii**). (**2**) SEM images of hollow PEMCs (**A**,**B**) and confocal images of PEMCs (**C**) with corresponding fluorescence profile. (**D**) The fluorescence arises from the complexation of Oregon-green labelled paclitaxel with the cyclodextrin the PEMC shell. Reprinted with permission from reference [265] copyright © 2013 American Chemical Society.

The antibody-functionalisation of the PEMC surface is also known. Recently, Ferrari et al. (2021) [257] encapsulated BSA into CaCO_3_ nanoparticles, with subsequent LbL of three bilayers of DS/pARG, followed by surface adsorption of anti-intracellular adhesion molecule-1 monoclonal antibody or donkey anti-mouse IgG secondary antibody. These LbL particles were uptaken by EA.hy926 endothelial cells and did not reduce cell viability, demonstrating the potential targeting ability of these antibody-functionalised particles. As aforementioned, biopolymer PEMCs are attractive in terms of their potential to mimic the extracellular matrix (ECM) as well as host bioactive molecules. Single- and multi-domain peptides (SDPs and MDPs, respectively) are emerging as materials for use in tissue engineering/regeneration applications within the development of nanofibrous scaffolds [258,259]. The incorporation of these bioactive-mimicking SDP/MDPs into PEMCs are of interest. For instance, MDPs based upon ECM proteins, which have neurite outgrowth promoting activity, may hold important applications in neuromuscular tissue regeneration [260]. Such programmed intrinsic bioactive responses, such as anti-inflammatory [261,262], osteogenic [263], and neural development responses [260], provide an efficient means of developing functional PEMC-based [38]/hydrogel scaffolds [219,259] to both guide cell growth and proliferation, as well as control cellular differentiation. Conjugating a functional molecule to the polyelectrolyte chain or varying the final layer of the multilayer are also options for the functionalisation of PEMCs. For instance, use of folic acid-conjugated chitosan upon DS/PRO-curcumin PEMCs [264] to improve mucoadhesive properties, and the conjugation incorporation of cyclodextrins [265,266] within multilayers and the PEMC interior. For instance, HA-conjugated β-cyclodextrin entities played host to the hydrophobic drug paclitaxel [265], as demonstrated in Figure 15. Polycationic cyclodextrins were also paired with ALG to act as antimicrobial agents with the capability to host guest molecules within the cyclodextrin and PEMC interior [267], demonstrating the PEMCs potential for use as a multifunctional, or even multicompartmental, drug delivery vehicle.

## 7. Conclusions and Future Outlook

Recent years have witnessed the versatile templating approach expand its scope of applications and it was successfully used for the architecting of biogenic-made materials. Providing one of the most accessible strategies for the synthesis of various porous materials with internal structure designed at the nanoscale, soft- and hard-templating became powerful tools for the production of capsules, beads, hybrid nano- and microparticles and their assemblies. Templating of such inherently complex and labile molecules as biopolymers is rather challengeable due to their nature, but it is worth the cost: the use of the biopolymers versus traditional synthetic analogues for the design of biomaterials manifests in excellent biocompatibility, biodegradability, no-to-low toxicity, the possibility of replicating the composition and structure of natural cellular microenvironments, and their intrinsic biofunctionality. Templating of biopolymers largely became possible due to the application of “bio-friendly” soft and hard templates, which not only do not require harsh dissolution agents for their elimination, but are also biocompatible and/or biodegradable themselves. In this context, it is hard to underestimate the importance of mesoporous carbonate templates, which are prone to dissolution at truly mild conditions and are fabricated in one step that can optionally be combined with the functionalisation of the particles via co-synthesis. Together with soft templating and coating of the crystal drugs, these approaches demonstrate the ability to generate biomaterials capable of controlled, optionally targeted, synergistic, and sustained multi-drug release, which can be driven by external or internal stimuli. From this point of view, the most promising clinical and biological applications are in (i) controlled drug delivery, (ii) sensing and bioimaging, (iii) theranostics, and (iv) tissue engineering and regenerative medicine. Among them, current drug delivery applications seem to be closest to the stage of lab-to-clinic transition. Other fascinating applications include cell patterning, functionalisation of biomaterial surfaces, functionalisation of the coating, etc.

Up-to-date reports in this field, including the first in vitro and in vivo studies, showed the high promise of biopolymer-based multilayer capsules and beads, made via templating, as biomaterials of the fourth generation. However, fundamental research on the mechanisms of interaction between these templated materials and biological cells, tissues and translational research are still needed. Remaining challenges in the field include the control over biomechanical properties, optimisation of synthetic procedures, approaches to enable up-scaled production and long-term storage of biopolymer-based particles, exploration of new cargo release mechanisms, and ways of particle functionalisation to decipher the interaction with other biomaterials and tissues. We believe that the next years of research will open opportunities for true biomedical translation of biopolymer-based multilayer capsules and beads made via the templating approach.

## Figures and Tables

**Table 1:** Summary of successfully fabricated nano- and microcapsules templated on porous templates as reported in literature. MF: melamine formaldehyde; NPs: nanoparticles; PS: polystyrene; ALG: alginate; CAR: carrageenan; CHI: chitosan; FC: fucoidan; HS: heparin sulphate; PR: protamine; pGLU: poly-L-glutamic acid; PLL: poly-L-lysine; PLL-pb: phenylboronic modified poly-l-lysine; and SP: spermidine.

Polyanion	Polycation	Template, Size	#Layers	References
ALG	CHI	MF, 2.1 μm	10	[47]
	PR	MF, 6.5 μm	8 and 16	[115]
	PLL	MF, 5.7 μm	5	[107]
	PLL-pb	Gold NPs, ~40 nm	4 and 8	[114]
CAR	CHI	SiO_2_-NH_2_, 100 nm	11	[116]
DS	CHI	Silica, 3 μm	14	[110]
		Silica, 330 nm	10	[117]
		Silica, 220 nm	8	[118]
	PR	MF, ~5 μm	8	[73,82]
HS	PR	Silica, 180 nm	6	[111]
	CHI	Silica, 220 nm	6	[96]
pGLU	CHI	MF, ~1 μm	10	[99]
		Silica, 330 nm	8	[112]
FC	CHI	PS, 90 nm	10	[108]
Pectin	CHI	SiO_2_-NH_2_, ~100 nm	7	[113]
DNA	SP	MF, 1.8 & 5.7 μm	5	[107]

**Table 2:** Summary of successfully fabricated nano- and microcapsules reported in literature. V-CaCO_3_: vaterite CaCO_3_; ALG: alginate; BSA: bovine serum albumin; CHI: chitosan; CS: chondroitin sulphate; CMC: carboxymethylcellulose; COL: collagen; DOX: doxorubicin; ELR: elastin-like recombinamer; FG2: basic fibroblast growth factor; GA: glutaraldehyde; HA: hyaluronic acid; HS: heparin sulphate; Hgb: hemoglobin; IgY: egg yolk immunoglobulin; LF: lactoferrin; MNP: magnetic nanoparticle; pARG: poly-L-arginine; pASP: poly(L-aspartic acid); pGLU: poly-L-glutamicacid; PLL: poly-L-lysine; pONT: poly-L-ornithine; PAH: poly(allylhydrochloride); PSS: poly(styrene sulfonate); and TA: tannic acid.

Polyanion	Polycation	Template, Size	#Layers	Reference
Polysaccharide-Based PEMCs				
CS	pARG	MnCO_3_, 4 μm	8	[142]
	PLL	V-CaCO_3_ pre-loaded with CS, 3–6 μm	10	[143]
		V-CaCO_3_, 7-9 μm	5	[79]
	PR	V-CaCO_3_ pre-loaded with PSS, 5 μm	4	[102]
		V-CaCO_3_, 7-9 μm	5	[79]
DS	pARG	V-CaCO_3_ pre-loaded with DEX, 3 μm	8	[144]
		V-CaCO_3_ pre-loaded with FG2	6–14	[145]
		V-CaCO_3_, 550 nm	6	[146]
	PR	V-CaCO_3_, 10 μm	7/8	[147]
		V-CaCO_3_, 7–9 μm	5	[79]
		V-CaCO_3_ pre-loaded with PSS,~4 μm	12	[148]
	CHI	V-CaCO_3_ pre-loaded with penicillin, ampicillin or ciprofloxacin, 5 μm	1-6	[136]
HA	PLL	V-CaCO_3_, 7–9 μm	5	[79]
		V-CaCO_3_, 5 μm	9	[149]
	COL	V-CaCO_3_ pre-loaded with BSA, 3–6 μm	12	[101]
HS	PLL	V-CaCO_3_, 7–9 μm	5	[79]
	CHI	V-CaCO_3_ pre-loaded with DOX, 4 μm	10	[150]
		V-CaCO_3_ coated with (PSS/PAH)_4_, 3–4 μm	9	[52]
	PR	V-CaCO_3_, 7–9 μm	5	[79]
	pARG	V-CaCO_3_ pre-loaded with HS, ~4 μm	4	[30]
		V-CaCO_3_, 3–5 μm	4	[151]
ALG	CHI	V-CaCO_3_ pre-loaded with CMC, 3–5 μm	10	[29]
	pARG	V-CaCO_3_ pre-loaded with PSS, 850 nm	4	[152]
ELR	CHI	V-CaCO_3_ pre-loaded with ovalbumin, 4 μm	4	[153]
Protein-based PEMCs				
TA	Pepsin and BSA	PLL-coated V-CaCO_3_, 3 μm	8	[154]
	BSA	PLL-coated V-CaCO_3_ pre-loaded with LF, 3 μm	8/16	[155]
	BSA	V-CaCO_3_ pre-loaded with BSA and MNPs, 3 μm	<6	[156]
GA-crosslinked BSA		MnCO_3_, 7.4 μm	10	[157]
GA-crosslinked Hgb		MnCO_3_, 5 μm	10	[158]
Polyamino acid-based PEMCs				
pASP	pARG	V-CaCO_3_ pre-loaded with pronase, 3–6 μm	7	[159]
pGLU	pONT	MS pre-loaded with DOX, 2 μm	8	[160]
	PLL	MS bare or pre-loaded with lysozyme or catalase, 2–4 μm	6	[161]
		V-CaCO_3_ pre-loaded with PLL or pGLU, 6 μm	7	[162]
		V-CaCO_3_ pre-loaded with IgY, 2–10 μm	10	[163]

**Table 3:** Summary of successfully fabricated nano- and microcapsules templated via drug crystal cores reported in literature. ALG: alginate; CHI: chitosan; DMPA: dimyristoylphosphatidic acid; DOX: doxorubicin; DS: dextran sulphate; HA: hyaluronic acid; HAS: human serum albumin; HS: heparin sulphate; GEL: gelatin; LS: lignosulfonate; PLL: poly-L-lysine; SF: silk fibronectin.

Polyanion	Polycation	Drug Crystal Core	Reference
ALG	CHI	Resveratol, ~200 nm	[227]
		Curcumin, 420 ± 17	[228]
	GEL	Naproxen, 11–20 um	[229]
Pectin	CHI	Indomethacin, ca. 200 nm	[230]
DS	CHI	Naproxen, 11–20 um	[229]
HA	-	DOX-coated cellulose nano-crystal, 134 ± 17 nm	[231]
HAS	DMPA	Ibuprofen, 15/36 um	[232]
HS	CHI	Insulin, ca 1 um	[233]
	PLL	Paclitaxel, 170–180 nm	[234]
		Camtothecin, <150 nm	[234]
SF	PLL	Dexamethasone, Side length 7.68 um, thickness of 750 nm	[231]
LS	CHI	Picloram, 1–4 um	[235]
